# Calibrating and Validating the MFI-UF Method to Measure Particulate Fouling in Reverse Osmosis

**DOI:** 10.3390/membranes13050535

**Published:** 2023-05-22

**Authors:** Mohanad Abunada, Nirajan Dhakal, William Z. Andyar, Yuke Li, Pamela Ajok, Noreddine Ghaffour, Jan C. Schippers, Maria D. Kennedy

**Affiliations:** 1 IHE-Delft Institute for Water Education, Water Supply, Sanitation and Environmental Engineering Department, Westvest 7, 2611 AX Delft, The Netherlands; 2 Faculty of Civil Engineering, Delft University of Technology, Stevinweg 1, 2628 CN Delft, The Netherlands; 3 Water Desalination and Reuse Center (WDRC), Biological and Environmental Science and Engineering (BESE) Division, King Abdullah University of Science and Technology (KAUST), Thuwal 23955-6900, Saudi Arabia

**Keywords:** reverse osmosis, particulate fouling, MFI-UF, calibration, linearity

## Abstract

This study aimed to calibrate and validate the MFI-UF method in order to ensure the accuracy of particulate fouling measurements in RO. Firstly, the MFI-UF calibration was examined using two solutions of standard particles (dextran and polystyrene). Two main criteria were investigated: (i) MFI-UF linearity with particle concentrations at both low and high ranges of fouling potential and (ii) the reproducibility of MFI-UF linearity. Dextran solutions showed a strong MFI-UF linearity over the entire range of measured MFI-UF. However, the linearity was not reproducible, and different batches of dextran prepared under the same conditions produced very variable results. For polystyrene solutions, the MFI-UF linearity was verified at the higher range of MFI-UF (>10,000 s/L^2^), while the MFI-UF at the lower range (<5000 s/L^2^) appeared to be underestimated. Secondly, MFI-UF linearity was investigated using natural (surface) water under a wide range of testing conditions (at 20–200 L/m^2^·h using 5–100 kDa membranes). Strong MFI-UF linearity was obtained over the entire range of measured MFI-UF (up to 70,000 s/L^2^). Thus, the MFI-UF method was validated to measure different levels of particulate fouling in RO. However, future research focusing on MFI-UF calibration is still required through the selection, preparation, and testing of heterogeneous mixtures of standard particles.

## 1. Introduction

The application of reverse osmosis (RO) membranes in water treatment has rapidly grown over the last few decades. Despite continuous advances, membrane fouling is still a major problem challenging the performance of this technology. Particulate fouling due to the deposition of particles and colloids onto the membrane is one of the fouling types experienced in RO systems. Membrane fouling can cause a decline in membrane permeability, which then requires higher operating pressures and more frequent membrane cleaning/replacement to maintain stable water production. Therefore, there is a real need for a reliable method to assess the particulate fouling potential of RO feeds in order to effectively control the operation of the RO systems.

The ASTM standard methods, i.e., the Silt Density Index (SDI) and Modified Fouling Index (MFI-0.45), are commonly used to assess the particulate fouling potential of RO feeds [1,2]. The MFI-0.45 has advantages over the SDI as (i) it is based on cake filtration which is assumed to be the dominant fouling mechanism in RO, (ii) it is proportional to the particle concentration in the feed water, and (iii) it can be corrected to reference testing conditions [3]. Nevertheless, the main drawback of the MFI-0.45 is the use of a membrane with a pore size of 0.45 um to simulate the fouling of the RO membrane. Hence, the measured MFI is too low to explain the fouling rates in RO, since small colloids (<0.45 um), which are likely responsible for RO membrane fouling, are not considered [4]. For this reason, the MFI-UF method, where the 0.45 um membrane was replaced by an ultrafiltration (UF) membrane to capture and assess smaller colloids, was developed [5,6,7,8].

The MFI-UF test was initially performed at constant pressure. However, in practice, most RO systems operate at a constant flux which is around 10–1000 times lower than the initial flux used during an MFI-UF test performed at constant pressure. As a result, since high filtration fluxes can result in cake compression, it was observed that the cake formed on MFI-UF membranes under constant pressure filtration may be more compressed than the cake formed on RO membranes, and thus, predicted particulate fouling may be overestimated [9]. Consequently, to more accurately assess particulate fouling in RO, the MFI-UF was developed to operate at a constant flux [9,10].

The measured MFI-UF also depends on the properties of the UF membrane used in the test, particularly on the molecular weight cut-off (MWCO). The lower the MWCO of the MFI-UF membrane, the smaller the membrane pore size and the more the particles that can be retained by the membrane, resulting in a higher MFI-UF value [10]. Furthermore, it was found in a previous study [11] that MFI-UF membranes with a lower MWCO have a lower surface porosity and thus smaller effective filtration area. This results in a higher local flux during the MFI-UF test and subsequently an overestimated MFI-UF value. To correct the effect of surface porosity, the study proposed correction factors from 0.4 to 1.0 for MFI-UF measured with 5–100 kDa membranes, respectively [11].

In addition, the MFI-UF value is highly dependent on the accuracy and reproducibility of the measurement set-up. The instrumentations that make up the MFI-UF set-up, particularly the (infusion) pump and pressure transmitter (explained in detail in Section 3.1), may drift with time due to malfunction, over-loading/extensive use, and environmental conditions, which may eventually result in errors in the outputs of these instruments. Moreover, the instrument errors may be exacerbated by improper test performance, due to (invisible) water leakage from the set-up and the existence of air bubbles [10], or because of a user-made error. Consequently, these errors may lead to an inaccurate MFI-UF value, eventually resulting in an inaccurate particulate fouling prediction.

Therefore, to ensure an accurate and consistent MFI-UF measurement, an MF-UF set-up should be calibrated using a standard (reference) calibration solution. For the MFI-0.45 method, the calibration could be performed using a standard solution of Formazine particles [2]. However, until now, no standard solution has been proposed or tested for the calibration of the MFI-UF method.

Moreover, in practice, the MFI-UF measures natural water (e.g., RO feed water) which usually includes a wide range of particles with different properties than those of standard calibration solutions. Therefore, in addition to the calibration of MFI-UF using a standard solution, the MFI-UF should be also validated using natural water. In both processes (i.e., calibration and validation), a linear correlation should be demonstrated between the MFI-UF value and particle concentration (as illustrated in Section 2).

Accordingly, the aim of this study was to calibrate and validate the MFI-UF for the accurate measurement of particulate fouling. For this purpose, the research objectives were:

(i) To examine the MFI-UF calibration using different calibration solutions of standard particles (dextran and polystyrene).
(ii) To validate the MFI-UF using natural (surface) water as a representative of the real water in RO systems.

## 2. MF-UF Theoretical Background

The MFI-UF is based on a cake/gel filtration mechanism [9]. At a constant flux, cake/gel filtration can be defined by the linear correlation of the transmembrane pressure (ΔP) and the filtration time (t), as shown in Equation (1).

(1) $$\Delta P=J\cdot \eta \cdot R_{m}+J^{2}\cdot \eta \cdot I\cdot t$$

where J is the flux rate, η is the feed water viscosity, $R_{m}$ is the clean membrane resistance, and I is the fouling index which describes the fouling potential of feed water. I is proportional to the product of the specific cake resistance (α) and particle concentration in the feed water (C), as shown in Equation (2).

(2) I=α·C

The specific cake resistance (α) can be defined based on the Carman–Kozeny equation [12], as shown in Equation (3), as a function of the cake porosity (ε), particle diameter (d), and particle density (ρ).

(3) $$\alpha =\frac{180\cdot \left(1-\varepsilon \right)}{\rho \cdot d^{2}\cdot \varepsilon^{3}}$$

**Figure 1:**
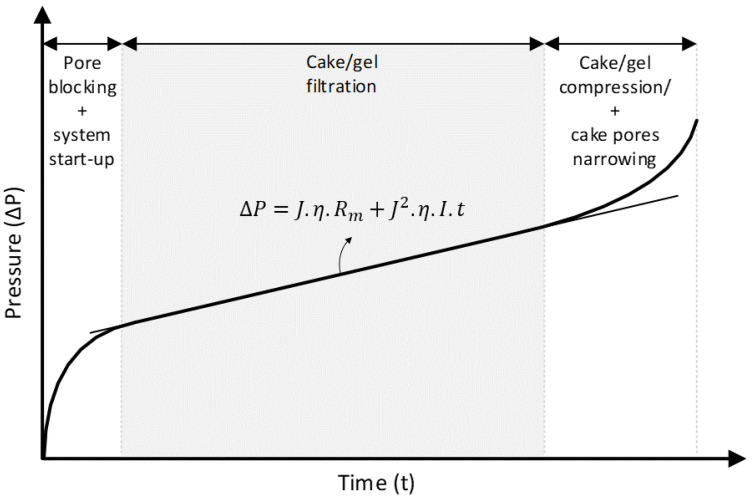
Typical filtration phases during the MFI-UF test performed at a constant flux.

The two parameters α and C are very difficult to measure accurately, especially for natural water. Therefore, the fouling index (I) can be determined experimentally through the MFI-UF test. During the MFI-UF test, transmembrane pressure increases over time in three sequential phases, (i) pore blocking (plus system start-up), (ii) cake/gel filtration, and (iii) cake/gel compression and cake pores narrowing, as shown in Figure 1. The value of I can be determined from the slope of the linear phase of cake/gel filtration using Equation (4).

(4) $$I=\frac{1}{J^{2}\cdot \eta }\cdot slope$$

The MFI-UF, by definition, is the value of I corrected to reference the testing conditions, as shown in Equation (5).

(5) $$MFI-UF=\frac{\eta_{20{}^{\circ}C}\cdot I}{2\cdot \Delta P_{o}\cdot A_{o}{}^{2}}$$

where $\Delta P_{o}$, $\eta_{20{}^{\circ}C},$ and $A_{o}$ are the reference pressure, water viscosity, and surface membrane area, respectively [3].

By combining Equations (2), (3) and (5), the MFI-UF can be then defined by Equation (6), which demonstrates (theoretically) a linear correlation between the MFI-UF and particle concentration.

(6) $$MFI-UF=\frac{90\cdot \eta_{20{}^{\circ}C}\cdot \left(1-\varepsilon \right)\cdot C}{\Delta P_{o}\cdot A_{o}{}^{2}\cdot \rho \cdot d^{2}\cdot \varepsilon^{3}}\;$$

## 3. Materials and Methods

### 3.1. MFI-UF Set-Up

**Figure 2:**
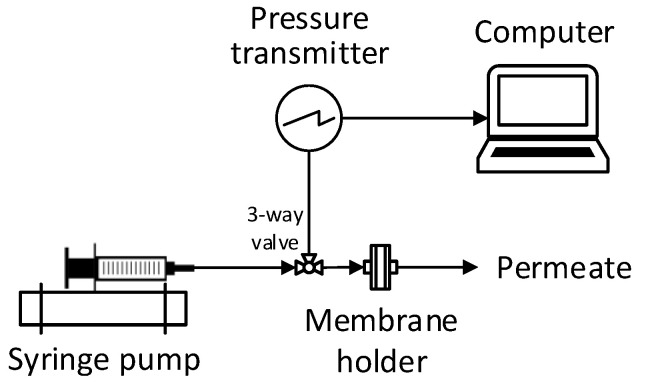
Scheme of the MFI-UF set-up at a constant flux filtration.

MFI-UF measurements were performed using the set-up schemed in Figure 2. The set-up simply consisted of three main items: an infusion syringe pump, pressure transmitter, and membrane holder (including the UF membrane). During the test, the water sample was delivered at a constant flow rate to the UF membrane. Simultaneously, the pressure transmitter recorded the transmembrane pressure (ΔP) over time (*t*) and transferred the data to a connected computer. Finally, the relationship between the ΔP and t was plotted (as shown in Figure 1), and then the MFI-UF value was calculated based on Equations (4) and (5).

Several MFI-UF set-ups were used to generate the experimental results for this study. The accuracy of the pumps and pressure transmitters was assured by following a quality control protocol (detailed in Supplemental Materials). Table 1 shows the accuracy and reproducibility (expressed by maximum % error) of all pumps and pressure transmitters used in this study.

### 3.2. MFI-UF Membranes

Flat-sheet polyethersulfone (PES) UF membranes with a molecular weight cut-off (MWCO) of 5, 10, and 100 kDa and a surface diameter of 25 mm were used (Biomax^®^, Millipore, MA, USA). All membranes were cleaned before use by filtering at least 100 mL of ultra-pure water (Milli-Q^®^, Millipore, MA, USA) to remove any preservation materials used during membrane production. Clean membrane resistance ($R_{m}$) was measured prior to each MFI-UF measurement, based on Equation (7), to ensure that the membranes were manufactured consistently and not damaged.

(7) $$R_{m}=\frac{\Delta P}{J\cdot \eta }\;$$

### 3.3. MFI-UF Calibration Using Standard Particle Solutions

MFI-UF calibration was examined using two solutions of different types of standard particles, dextran and polystyrene, as representative of polymeric and well-defined particles, respectively. The applied testing conditions are detailed below and summarized in Table 2.

Dextran with a molecular weight of 150 kDa was used (Sigma-Aldrich, St. Louis, MO, USA) and was supplied in a powder form. Dextran solutions with serial concentrations (Table 2) were prepared by dissolving the dextran in a 0.05 mol/L potassium phosphate buffer solution of pH 7. The buffer solution was prepared by dissolving 3.15 g of KH_2_PO_4_ and 4.67 g of K_2_HPO_4_ in 1 L of ultra-pure water (Milli-Q^®^, Millipore, MA, USA).

Polystyrene particles had a nominal size of 25 nm (Bangs Laboratories, Fishers, IN, USA). The supplied polystyrene was suspended in deionized water with a concentration of 10%. The suspension also included 0.5% sodium dodecyl sulfate (to stabilize the particles) and 0.1% sodium azide (to inhibit bacterial growth). Polystyrene solutions of different concentrations (Table 2) were prepared by adding ultra-pure water (Milli-Q^®^, Millipore, MA, USA) to the supplied stock suspension.

The suitability of dextran and polystyrene solutions for MFI-UF calibration was examined based on two main criteria:

1. The verification of MFI-UF linearity at both the lower and higher range of particle concentration. For RO applications, it is important that the calibration/linearity range covers the MFI-UF of RO feed water. Based on previous work (Abunada et al., 2023), the MFI-UF values of different RO feed waters measured at the same testing conditions shown in Table 2 (at 100 L/m^2^·h using 100 kDa membrane) were in the range of 550–1150 s/L^2^. However, this range may be different for other types of RO feed water.
2. The reproducibility of MFI-UF linearity under the same testing conditions (i.e., at the same flux and same MFI-UF membrane MWCO).

### 3.4. MFI-UF Validation Using Natural Water

MFI-UF validation was investigated by verifying the linearity of the MFI-UF as a function of the particle concentration using natural surface water to simulate the real water in the RO systems in practice. For this purpose, several batches of canal water (CW) were collected (from Delft, the Netherlands). The quality of the CW varied based on the time of collection (season, month, and even the time of day), where the turbidity ranged from 1.5 to 3.0 NTU, TOC from 11 to 18 mg/L, and EC from 600 to 800 uS/cm. Tap water was used to make dilutions of the CW to eliminate any effect of pH variation, as both CW and tap water had more or less the same pH. Accordingly, all prepared CW dilutions had similar pH (7.9–8.1). Since tap water is not a particle-free solution (i.e., it also contains particles), it was filtered through a 10 kDa membrane before being used to dilute the CW, in order to avoid introducing additional particles into the diluted CW samples. Diluted CW samples were prepared in two ranges of concentrations, low and high (as shown in Table 3), to simulate the water with both low and high particle concentrations such as the RO feed and raw water, respectively.

In principle, the MFI-UF linearity should be verified at the flux which is typically applied in RO systems, i.e., in the range from 10 to 35 L/m^2^·h (DOW, 2011). However, at such a low flux range, the MFI-UF test may be very lengthy. Hence, the MFI-UF can be measured at higher flux rates and then extrapolated linearly to the actual RO flux [13]. Therefore, the MFI-UF linearity was investigated at a low flux of 20 L/m^2^·h (similar to the RO flux) as well as at higher flux rates of 100 and 200 L/m^2^·h.

In addition, since the size of particles depositing onto RO membranes is unknown, the MFI-UF can be typically measured using UF membranes with a range of MWCOs [10]. Hence, the MFI-UF linearity was investigated using different UF membranes with MWCO of 5, 10, and 100 kDa.

The testing conditions applied to assess the MFI-UF linearity using CW are summarized in Table 3.

## 4. Results and Discussion

### 4.1. MFI-UF Calibration Using Standard Solutions

#### 4.1.1. Dextran Particle Solution

##### Linearity Verification

**Figure 3:**
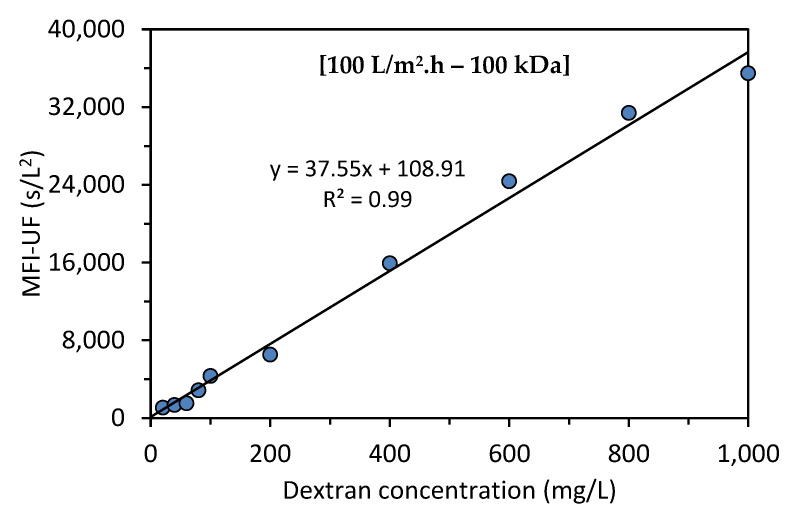
Relationship between the MFI-UF and dextran concentration.

Figure 3 shows the relationship between the MFI-UF and dextran concentration at 100 L/m^2^·h using a 100 kDa membrane. As can be observed, the calibration curve was linear over the entire range of dextran concentrations, with $R^{2}$ > 0.99.

##### Reproducibility

To verify the reproducibility of the MFI-UF calibration line demonstrated in Figure 3, the MFI-UF measurements were repeated at the same conditions (at 100 L/m^2^·h using 100 kDa membrane) using newly prepared dextran samples (prepared by the same stock dextran). The relationship between the MFI-UF and the dextran concentration of the new samples was also linear ($R^{2}$ = 0.93). However, the slope of the calibration line obtained with the different batches of dextran samples varied by around 70%, which indicated that the calibration line obtained with dextran solutions was not reproducible.

**Figure 4:**
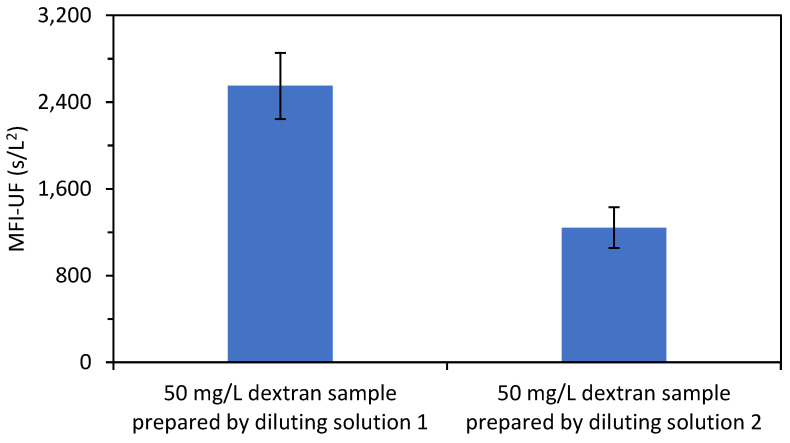
MFI-UF of dextran samples (50 mg/L) prepared with two diluting buffer solutions (with the MFI-UF measured in triplicate).

The reason that the MFI-UF calibration line was not reproducible with the dextran solutions was assumed to be attributed to the diluting buffer solution (potassium phosphate buffer solution) used to prepare the dextran samples. To confirm this, the MFI-UF was measured using samples of dextran (50 mg/L) prepared with two diluting buffer solutions which were prepared based on the same procedure explained in Section 3.3. As shown in Figure 4, the variation in the MFI-UF of the dextran samples prepared with the same diluting solution was 12–15% (with the MFI-UF measurements performed in triplicate for each diluting solution). However, the variation in the average MFI-UF of the dextran samples prepared with different diluting solutions was more than 100%.

This result could be attributed to the structure of the dextran polymer chains (i.e., the type, degree, and length of branching) which could be sensitive to any slight variation in the chemical stability of the prepared diluting buffer solution. Another reason could be due to the sensitivity of dextran polymers to the stirring conditions (i.e., shear forces) during the preparation of the dextran solutions. Consequently, the size range of dextran particles might have been different for each of the prepared dextran samples shown in Figure 4. Hence, since the MFI-UF is highly dependent on particle size (Equation (6)), a different range of MFI-UF values (and thus a different calibration line) could be obtained when very small changes in the diluting buffer solution exist.

#### 4.1.2. Polystyrene Particle Solution

##### Linearity Verification

**Figure 5:**
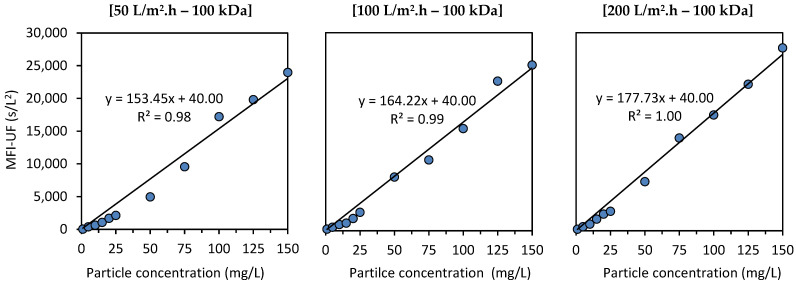
Relationship between the MFI-UF and polystyrene concentration.

Figure 5 shows the relationship between the MFI-UF and the concentration of polystyrene particles at 50, 100, and 200 L/m^2^·h using a 100 kDa membrane. The intercepts of the calibration lines were set to 40 s/L^2^, which is the average MFI-UF value of the diluting blank solution (ultra-pure water) at these testing conditions. As observed, the fit of the calibration line to the measured MFI-UF values was similar in all cases; the higher MFI-UF values (> ~10,000 s/L^2^) of higher polystyrene concentration samples (50–150 mg/L) showed a good fit with the regression line, while the MFI-UF values obtained at the lower particle concentration range (1–25 mg/L) showed a poor fit and appeared to be underestimated. The observation of this trend may be due to two reasons, as explained below.

One possible reason for this trend (shown in Figure 5) can be attributed to the surfactant portion in the prepared polystyrene samples (sodium dodecyl sulfate was present in the supplied polystyrene suspension as a surfactant to stabilize the particles). The surfactant concentration in the lower polystyrene concentration samples might have been too low to stabilize the particles. As a result, the polystyrene particles in the lower concentration samples could aggregate into larger particles, and hence a lower (underestimated) MFI-UF was obtained (based on Equation (6)). To verify this hypothesis, the zeta potential (ZP) of the polystyrene samples was measured using a zetasizer (Malvern, Nano-ZS). For all polystyrene samples (both of lower and higher polystyrene concentration), the ZP was similar, with a value of −61(±1) mV (except for the polystyrene sample of 1 mg/L where the ZP was −50 mV). This indicated that the required surfactant portion was based on the particle–surfactant ratio (i.e., the lower the particle concentration, the lower the surfactant concentration required to keep the particles stabilized), and thus, the surfactant portion was sufficient even in the samples with a lower polystyrene concentration. Therefore, the aggregation of polystyrene particles might not be the reason for the underestimated MFI-UF values observed at lower particle concentrations shown in Figure 5.

**Figure 6:**
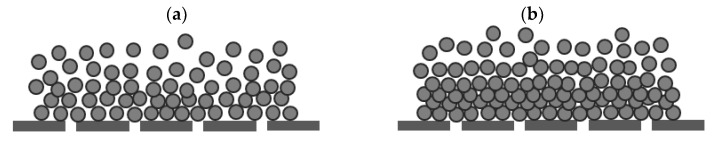
Hypothesized illustration of the cake formed by polystyrene particles in case of a (**a**) lower particle concentration and (**b**) higher particle concentration.

Another reason can be attributed to the inert nature of polystyrene particles resulting in the restriction of their attachment to the MFI-UF membrane surface as well as to each other during MFI-UF testing. This effect of the inert nature of polystyrene could be further enhanced by several factors, i.e., (i) the presence of surfactant which could keep the polystyrene particles stabilized and unattached, (ii) the lateral water flow streams inside the MFI-UF membrane holder which could sweep the particles from the membrane surface, and (iii) the low density of polystyrene particles. Due to these factors, at lower polystyrene concentrations, a porous cake might have been formed above the MFI-UF membrane surface, as shown in Figure 6a. Consequently, the MFI-UF values at lower particle concentrations were underestimated (below the calibration line). On the other hand, at higher polystyrene concentrations, the load of particles was higher, which could subsequently overcome the aforementioned factors by pushing and holding the particles on the membrane surface, forming a more compacted cake, as shown in Figure 6b. Therefore, the MFI-UF values at the higher range were fitted well by the calibration line (i.e., not underestimated as the case at the lower range).

The above explanation also clarifies the improvement of the fit of the calibration line at the lower range when the flux was higher (i.e., the MFI-UF values at the lower range were less underestimated when the flux was higher). This is because the higher the flux, the higher the permeation force acting on particles toward the membrane surface, which subsequently reduced the effect of the factors mentioned in the above explanation.

##### Reproducibility

The reproducibility of the MFI-UF calibration line obtained with polystyrene solutions was examined at a flux of 100 L/m^2^.h using a 100 kDa membrane using newly prepared batches of polystyrene particles. The relationship between the MFI-UF and the concentration of the new polystyrene particles was similar to that demonstrated in Figure 5, where the slopes of the calibration lines obtained by the different batches of polystyrene samples were similar, with only a 7% deviation.

However, despite the reproducibility of the MFI-UF calibration line obtained with polystyrene solutions, the reproducibility of the calibration line appeared to be similar regardless of the applied flux rate. Based on the results shown in Figure 5, the slopes of calibration lines were similar (with only a 7% variation), although they were obtained at different flux rates (50, 100, and 200 L/m^2^·h). In principle, increasing the flux directly impacts the arrangement of particles in the cake and simultaneously causes cake compression, which eventually results in a less-porous cake and, thus, a higher MFI-UF value [14]. However, the effect of the flux might be minor in the case of the tested polystyrene solutions. This is because the used polystyrene particles are monodisperse spheres, and, hence, particle rearrangement in the cake is limited, even when the flux was increased from 50 to 200 L/m^2^·h. In addition, since polystyrene particles are rigid [15], they are not expected to be strongly compressed as the flux is increased. Consequently, cake porosity and thus the measured MFI-UF values were similar at different flux rates, which resulted in similar calibration lines. This result suggests that the MFI-UF calibration with polystyrene solution may not be able to detect errors in pump flow (if any), as the MFI-UF of a polystyrene sample would be similar even if the flux was different due to a pump error. However, using a heterogenous mixture of polystyrene particles (with different particle sizes and shapes) may improve the degree of the rearrangement and compressibility of polystyrene particles in the cake and overcome the limitation of calibration mentioned above. Nevertheless, this needs further investigation.

### 4.2. MFI-UF Linearity Using Natural Water (Canal Water)

**Figure 7:**
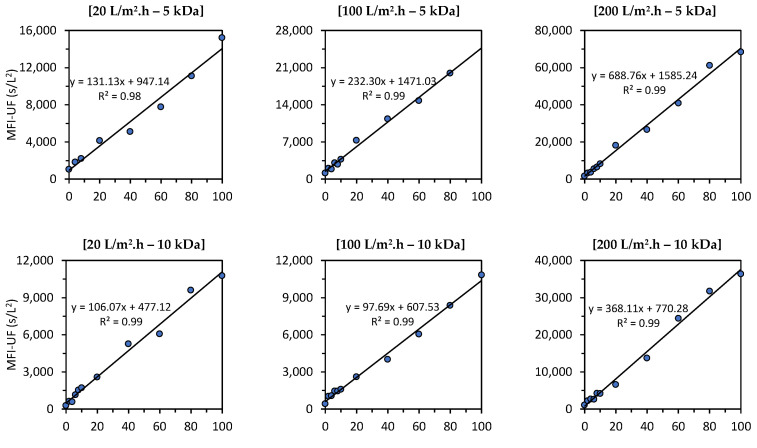

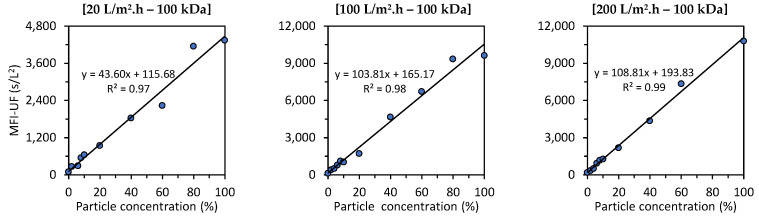
Relationship between the MFI-UF and particle concentration in canal water at various testing conditions; at 20, 100, and 200 L/m^2^·h using 5, 10, and 100 kDa membranes (the raw canal water used to prepare the dilutions was different at each testing condition; turbidity = 1.5–3.0 NTU, TOC = 11–18 mg/L and EC = 600–800 uS/cm).

Figure 7 shows the relationship between the MFI-UF and the particle concentration in canal water (CW) under different testing conditions (at flux rates of 20, 100, and 200 L/m^2^·h using 5, 10, and 100 kDa membranes). As explained in Section 3.4, the raw CW used to prepare the CW dilutions was different for each testing condition, since the raw CW was collected at different times of the year. This means that the water quality for each testing condition was different (turbidity = 1.5–3.0 NTU, TOC = 11–18 mg/L, and EC = 600–800 uS/cm). Therefore, the effect of the flux and membrane MWCO on the MFI-UF values (and thus on the slopes of linear relationships) in Figure 7 cannot be compared. For instance, for the 10 kDa membranes, the MFI-UF values measured at 20 L/m^2^·h were close to the corresponding MFI-UF values measured at a higher flux of 100 L/m^2^·h. This is because the quality of raw CW used at 20 L/m^2^·h was lower than that at 100 L/m^2^·h (e.g., the TOC was 16 and 11.2 mg/L for the raw CW used at 20 and 100 L/m^2^·h, respectively).

As observed at all testing conditions, the relationship between the MFI-UF and the particle concentration was linear, with $R^{2}$ > 0.97, regardless of the flux, membrane MWCO, and water quality tested. This result verified that the MFI-UF method is valid (according to Equation (6)) and can be used to measure the particulate fouling potential at a wide range of testing conditions (at fluxes of 20–200 L/m^2^·h using 5, 10, and 100 kDa membranes). In addition, the strong correlation between the MFI-UF and particle concentration also confirmed the robustness of the MFI-UF method to detect the variation in particulate fouling potential due to any changes in particle concentration in RO feed water.

Based on Figure 7, the MFI-UF linearity range was verified up to around 70,000 s/L^2^ (at 200 L/m^2^·h using a 5 kDa membrane). However, it is expected that the MFI-UF linearity could be extended to higher levels of MFI-UF in cases where other types of water (with higher particle concentrations) are used. Nevertheless, the observed linearity ranges (Figure 7) were confirmed to cover the MFI-UF levels among different RO plants measured in previous works [13,16].

Nevertheless, the MFI-UF was validated using fresh water (canal water), while the RO systems in practice may also be used to treat saline water, i.e., brackish groundwater and seawater. Regarding the application of the MFI-UF for saline water, Boerlage [17] found that an increase in the salinity of the feed water (such as the case in seawater) can affect the cake resistance and thus the MFI-UF. An increase in salinity can initially compress the double layer around the particles which results in an increase in the cake resistance and thus a higher MFI-UF value. Once the increase in salinity is above the critical concentration of coagulation, particles start to aggregate into larger sizes, leading to less cake resistance and, thus, a lower MFI-UF value. Hence, if seawater is used to validate the MFI-UF (i.e., instead of fresh surface water), the diluted seawater samples will have different salinity, which will affect the particle/cake properties as mentioned above. However, based on the MFI-UF principle (Equation (6)), the MFI-UF method can be validated if a linear relationship exists between the MFI-UF and particle concentration, assuming no change in particle/cake properties (i.e., particle size and density and cake porosity). Therefore, the MFI-UF of diluted seawater samples should be corrected to eliminate the effect of salinity [17]. Consequently, and based on Equation (6), the corrected MFI-UF will then only be a function of particle concentration, as is the case in the surface canal water used in this work.

## 5. Conclusions

This study aimed to calibrate and validate the MFI-UF for the accurate measurement of particulate fouling in RO.

The MFI-UF calibration was examined using two solutions of standard particles (150 kDa dextran and 25 nm polystyrene particles). The calibration was examined based on: (i) the verification of MFI-UF linearity at both the lower and higher range of MFI-UF and (ii) the reproducibility of MFI-UF linearity under the same testing conditions.

For dextran solutions, the relationship between the MFI-UF and particle concentration was strongly linear over the entire range of MFI-UF ($R^{2}\approx$ 1). However, the calibration line was not reproducible with different batches of dextran solution prepared under the same conditions. The reason could be attributed to the dextran polymers, which might be sensitive to the very slight variation in sample preparation (i.e., a slight variation in the stirring conditions and/or chemical stability), which could consequently result in different particle sizes and thus different MFI-UF values when dextran samples (of the same concentration) were prepared with different diluting solutions. Therefore, the dextran solution was not deemed suitable for MFI-UF calibration.

For polystyrene solutions, a linear relationship between the MFI-UF and particle concentration was verified at the higher range of MFI-UF (i.e., MFI-UF > 10,000 s/L^2^), while the MFI-UF values at the lower range appeared to be underestimated. The explanation of this trend could be attributed to the inert nature of polystyrene particles which could restrict their attachment to the MFI-UF membrane surface as well as to each other during the MFI-UF test. Consequently, at lower concentration (i.e., load) of polystyrene particles, a more porous cake might have formed on the MFI-UF membranes and thus lower (i.e., underestimated) MFI-UF values were obtained. In addition, the calibration lines obtained for polystyrene solutions were similar over a wide range of flux rates (50–200 L/m^2^.h). This was attributed to the fact that the tested polystyrene particles were hardly rearranged or compressed even when the flux was increased. Therefore, the cake porosity and thus the measured MFI-UF values were similar at different flux rates. This result indicated that the polystyrene particle solutions may not be able to detect errors in the pump (i.e., the MFI-UF of a polystyrene sample would be similar even if the flux changed due to any error in the pump flow).

The MFI-UF validation was investigated by verifying the linearity of MFI-UF using natural water (canal water) under a wide range of testing conditions (i.e., at a flux of 20, 100, and 200 L/m^2^·h using 5, 10, and 100 kDa membranes). At all testing conditions, the relationship between the MFI-UF and the particle concentration was strongly linear at both the lower and higher range of MFI-UF ($R^{2}$ > 0.97). The verified linearity ranges covered MFI-UF levels among different RO plants (measured in previous works).

In conclusion, the MFI-UF method was validated for measuring particulate fouling under a wide range of testing conditions. On the other hand, further research is still required to examine the MFI-UF calibration using more suitable solutions of standard particles. Based on the results of this study, the solution of polystyrene 25 nm standard particles is considered promising as it is stable and reproducible (compared to the dextran solution). However, further investigation is needed to select, prepare, and test a suitable heterogeneous mixture of polystyrene particles (with different particle sizes and shapes) to overcome the limitations of the calibration mentioned above.

## Figures and Tables

**Table 1:** Accuracy and reproducibility of pumps and pressure transmitters (expressed by maximum % error).

Instrument	Model, Manufacturer	Accuracy	Reproducibility
Infusion pump	PHD ULTRA^TM^ HPSI, Harvard Apparatus	5% < set flow	1%
	PHD ULTRA^TM^ standard, Harvard Apparatus	5% < set flow	1%
Pressure transmitter	Cerabar PMC51, Endress+Hauser	±1%	0.5%
	PXM409, Omega	±1%	0.5%

**Table 2:** Testing conditions applied to assess the MFI-UF linearity using dextran and polystyrene solutions.

Standard Particles Used in Feed Solution	Concentrations	Flux	MFI-UF Membrane
Dextran (150 kDa)	Lower range: 20, 40, 60, 80, and 100 mg/LHigher range: 200, 400, 600, 800, and 1000 mg/L	100 L/m^2^·h	100 kDa
Polystyrene (25 nm)	Lower range: 1, 5, 10, 15, 20, and 25 mg/LHigher range: 50, 75, 100, 125, and 150 mg/L	50, 100, and 200 L/m^2^·h	100 kDa

**Table 3:** Testing conditions applied to assess the MFI-UF linearity using CW.

Feed Solution	Concentration *	Flux	MFI-UF Membrane
Canal water (CW)	Lower range: 0, 2, 4, 6, 8, and 10% Higher range: 20, 40, 60, 80, and 100%	20, 100, and 200 L/m^2^·h	5, 10, and 100 kDa

* For example, a 10% sample refers to a mixture of 10% CW and 90% diluting solution (prefiltered tap water). A 0% sample refers to 0% CW, i.e., only the diluting solution (prefiltered tap water).

## Data Availability

Not applicable.

## Supplementary Materials

The following supporting information can be downloaded at: https://www.mdpi.com/article/10.3390/membranes13050535/s1, Supplementary File: Quality control protocol to ensure the accuracy of the MFI-UF set-up instruments.

## Nomenclature

MFI-UF	Modified fouling index—ultrafiltration {s/L^2^}
I	Fouling index {1/m^2^}
J	Flux {L/m^2^·h or m^3^/m^2^·s}
ΔP	Transmembrane pressure {bar or Pa}
$R_{m}$	Clean membrane resistance {1/m}
η	Feed water viscosity {N·s/m^2^}
t	Filtration time {s, min, h, day, or month}
C	Particle concentration {mg/L}
α	Specific cake resistance {m/kg}
ε	Cake porosity {-}
*ρ*	Particle density {kg/m^3^}
d	Particle diameter {m}
$\Delta P_{o}$	Reference transmembrane pressure {= 2 bar or 200 kPa}
$\eta_{20{}^{\circ}C}$	Reference water viscosity at 20 °C {= 0.001002 N·s/m^2^}
$A_{o}$	Reference MFI membrane surface area {= 0.00138 m^2^}

## References

[1] (2014). Standard Test Method for Silt Density Index (SDI) of Water.

[2] (2015). Standard Test Method for Modified Fouling Index (MFI-0.45) of Water.

[3] Schippers J.C., Verdouw J. (1980). The modified fouling index, a method of determining the fouling characteristics of water. Desalination.

[4] Schippers J.C., Folmer H.C., Verdouw J., Scheerman H.J. (1985). Reverse osmosis for treatment of surface water. Desalination.

[5] Boerlage S.F.E., Kennedy M.D., Aniye M.P., Abogrean E., Tarawneh Z.S., Schippers J.C. (2003). The MFI-UF as a water quality test and monitor. J. Membr. Sci..

[6] Boerlage S.F.E., Kennedy M.D., Aniye M.P., Abogrean E.M., Galjaard G., Schippers J.C. (1998). Monitoring particulate fouling in membrane systems. Desalination.

[7] Boerlage S.F.E., Kennedy M.D., Bonne P.A.C., Galjaard G., Schippers J.C. (1997). Prediction of flux decline in membrane systems due to particulate fouling. Desalination.

[8] Boerlage S.F.E., Kennedy M.D., Dickson M.R., El-Hodali D.E.Y., Schippers J.C. (2002). The modified fouling index using ultrafiltration membranes (MFI-UF): Characterisation, filtration mechanisms and proposed reference membrane. J. Membr. Sci..

[9] Boerlage S.F.E., Kennedy M., Tarawneh Z., De Faber R., Schippers J.C. (2004). Development of the MFI-UF in constant flux filtration. Desalination.

[10] Salinas-Rodríguez S.G., Amy G.L., Schippers J.C., Kennedy M.D. (2015). The modified fouling index ultrafiltration constant flux for assessing particulate/colloidal fouling of RO systems. Desalination.

[11] Abunada M., Dhakal N., Andyar W.Z., Ajok P., Smit H., Ghaffour N., Schippers J.C., Kennedy M.D. (2022). Improving MFI-UF constant flux to more accurately predict particulate fouling in RO systems: Quantifying the effect of membrane surface porosity. J. Membr. Sci..

[12] Carman P.C. (1938). Fundamental principles of industrial filtration (A critical review of present knowledge). Trans. Instn Chem. Engrs..

[13] Salinas-Rodríguez S.G., Kennedy M.D., Amy G.L., Schippers J.C. (2012). Flux dependency of particulate/colloidal fouling in seawater reverse osmosis systems. Desalination Water Treat..

[14] Salinas-Rodriguez S.G. (2011). Particulate and Organic Matter Fouling of Seawater Reverse Osmosis Systems: Characterization, Modelling and Applications. Dissertation Thesis.

[15] Kutscher H.L., Chao P., Deshmukh M., Singh Y., Hu P., Joseph L.B., Reimer D.C., Stein S., Laskin D.L., Sinko P.J. (2010). Threshold size for optimal passive pulmonary targeting and retention of rigid microparticles in rats. J. Control. Release.

[16] Abunada M., Dhakal N., Gulrez R., Ajok P., Li Y., Abushaban A., Smit H., Moed D., Ghaffour N., Schippers J.C. (2023). Prediction of particulate fouling in full-scale reverse osmosis plants using the modified fouling index – ultrafiltration (MFI-UF) method. Desalination.

[17] Boerlage S.F.E., Kennedy M., Aniye M.P., Schippers J.C. (2003). Applications of the MFI-UF to measure and predict particulate fouling in RO systems. J. Membr. Sci..

