# Supplementary material for: Calibrating and Validating the MFI-UF Method to Measure Particulate Fouling in Reverse Osmosis
Source: Membranes (Basel). 2023 May 22;13(5):535. doi: 10.3390/membranes13050535 (PMC10222867; doi:10.3390/membranes13050535)
Supplement: Supplementary file 1 [file membranes-13-00535-s001.zip › membranes-2402018-supplementary.pdf]

Quality control protocol to ensure the accuracy of the MFI-UF set-up instruments

In order to ensure the quality of MFI-UF measurements, the accuracy and reproducibility of the pumps and pressure transmitters used in the MFI-UF set-up were checked frequently during this study, as explained below.

Pump flow accuracy was checked at different flow rates in the range of 6.93 – 69.30 mL/h, which is equivalent to the flux range applied during this study (20-200 $L/m^2.h$). At each testing flow rate, the infused water was collected and weighed using an electronic balance. Accordingly, the water volume and thus water flow rate was measured over time. For the pumps used in this study, the maximum difference between the actual and set pump flow at the entire testing range (20-200 $L/m^2.h$) was < 5%, which was considered acceptable. In addition, the average flow rate of the pumps was very reproducible, where the variation did not exceed 1%.

The accuracy of pressure transmitter was checked in the pressure range of 20-5000 mbar, which is the transmembrane pressure range mostly observed during MFI-UF tests performed in this study (at the flux of 20-200 $L/m^2.h$ using 5-100 kDa membranes). Pressure transmitter was connected to a pressure vessel, where the pressure was set constant using a pressure regulator. The transmitter readings were compared with the outputs of a calibrated manometer connected also to the pressure vessel. The maximum difference between the transmitter readings and the manometer outputs was always less than ±1%, while the variation in the average transmitter readings was less than 0.5%.
